# The Pathobiology of Behavioral Changes in Multiple System Atrophy: An Update †

**DOI:** 10.3390/ijms25137464

**Published:** 2024-07-07

**Authors:** Kurt A. Jellinger

**Affiliations:** Institute of Clinical Neurobiology, Alberichgasse 5/13, 1150 Vienna, Austria; kurt.jellinger@univie.ac.at; Tel./Fax: +43-1-5266534

**Keywords:** multiple system atrophy, behavioral features, anxiety, compulsive disorders, neuroimaging, brain network dysfunctions

## Abstract

While cognitive impairment, which was previously considered a red flag against the clinical diagnosis of multiple system atrophy (MSA), is a common symptom of this rare neurodegenerative disorder, behavioral disorders are reported in 30 to 70% of MSA patients. They include anxiety, apathy, impaired attention, compulsive and REM sleep behavior disorders (RBD), and these conditions, like depression, are early and pervasive features in MSA, which may contribute to disease progression. Despite changing concepts of behavioral changes in this synucleinopathy, the underlying pathophysiological and biochemical mechanisms are poorly understood. While specific neuropathological data are unavailable, neuroimaging studies related anxiety disorders to changes in the cortico-limbic system, apathy (and depression) to dysfunction of prefrontal–subcortical circuits, and compulsive behaviors to impairment of basal ganglia networks and involvement of orbito-frontal circuits. Anxiety has also been related to α-synuclein (αSyn) pathology in the amygdala, RBD to striatal monoaminergic deficit, and compulsive behavior in response to dopamine agonist therapy in MSA, while the basic mechanisms of the other behavioral disorders and their relations to other non-motor dysfunctions in MSA are unknown. In view of the scarcity of functional and biochemical findings in MSA with behavioral symptoms, further neuroimaging and biochemical studies are warranted in order to obtain better insight into their pathogenesis as a basis for the development of diagnostic biomarkers and future adequate treatment modalities of these debilitating comorbidities.

## 1. Introduction

Multiple system atrophy (MSA) is an adult-onset and lethal neurodegenerative disorder of uncertain etiology, and is clinically characterized by various combinations of autonomic (urogenital and cardiovascular) failure, levodopa poorly responsive Parkinsonism, motor, non-motor, and cerebellar symptoms [1,2,3]. The pathological hallmarks of this oligodendroglio-neuronal synucleinopathy are α-synuclein (αSyn) immunoreactive glial cytoplasmic inclusions (GCI) mainly in oligodendroglia, and their presence is mandatory for the diagnosis of definite MSA [4] and neuronal cytoplasmic/nuclear inclusions. The GCI burden correlates with the neurodegeneration of the striatonigral and/or olivocerebellar systems and diffuse demyelination [5] that clinically manifest as Parkinsonian (MSA-P) and cerebellar (MSA-C) variants [6], although there is a broad spectrum of mixed and atypical variants of this disorder [7]. MSA is a rare disease with an estimated incidence of 0.6–0.7/100,000 person years and a prevalence of 1.9–4.9/100,000 [1]. MSA-P accounts for 70–80% of cases in the western world, whereas MSA-C is more frequent in Asian populations, probably due to genetic and environmental factors [3]. While cognitive impairment, often progressing to dementia, is a common non-motor symptom in MSA [8,9,10,11], accumulating evidence suggests that behavioral disorders are also frequent in non-demented patients with MSA [1].

Behavioral disorders, according to the definition of the 5th edition of the American Psychiatric Association Diagnostic and Statistical Manual DSM-5-TR, are summarized in Table 1. They occur in early stages of MSA, sometimes even before the appearance of classical motor symptoms [12], but are also associated with advanced disease stages [13]. Anxiety, agitation, apathy, impulse control disorders, and REM sleep behavioral disorder (RBD) are the most common behavioral changes in MSA [13,14,15]. Obsessive compulsive disorders (OCD) may also occur, but these are less common [16].

Living with either MSA and/or severe behavioral symptoms, often mixed with depression and cognitive impairment, is difficult and impairs the quality of life of patients and caregivers, since they are fundamental for the progression of the disease, and should be considered as part of its diagnosis and treatment. Yet, there is little information about the pathophysiological mechanisms of behavioral changes in MSA and their relations to other manifestations of the disease. This article, based on a systematic literature research of PubMed, Google Scholar and Cochrane Library until May 2024, aims to explain the relations between MSA and behavioral disturbances, their epidemiology, basic clinical features, neuroimaging findings, pathogenic factors, and current treatment options.

## 2. Clinical Features of Behavioral Symptoms in MSA

Anxiety is defined as a neuropsychiatric disorder characterized by nervousness and loss of concentration, due to the anticipation of impending danger. At baseline, anxiety may be mild, but its severity is associated with increased disease duration and severity [13,17]. Frontal behavioral changes in MSA, most commonly apathy and inflexibility, are also associated with disease severity, anxiety, and depression, causing deterioration of quality of life [18]. 

Patients with MSA-P show deficits in executive function and higher anxiety (and depression) scores than healthy controls [19]. Behavioral impairment in MSA compromises females more than males [20].

Impulse control disorders are a group of symptoms that, according to the DSM-5, are characterized by destructive behaviors related to impulse control, including problems with self-control over one’s emotions and behaviors [21]. The co-occurrence of multiple compulsive behaviors with anxiety [22] and selective impairment of attentional function has been reported in MSA, but the underlying mechanisms are still unclear [23].

Isolated RBD, a parasomnia, was recently recognized as a risk factor for MSA. It is estimated that 4–5% of patients with idiopathic RBD will develop MSA [24], and approximately 30–40% of those with MSA exhibit RBD symptoms prior to the onset of the disease. Most of them reported that RBD was the initial symptom of MSA [25,26]. In more than half of the MSA patients, symptoms of RBD occurred before the onset of motor deficits [14]. Although MSA frequently accompanies REM sleep without atonia (RWA), most of the RBD symptoms occur just prior to or at the onset of MSA and then disappear within a short period [27]. Their onset as a presenting symptom of MSA during the premotor period does not differ between the subtypes of MSA [28].

## 3. Epidemiology of Behavioral Changes in MSA

According to available data, up to 70% of MSA patients show various forms and degrees of behavioral changes in one or several domains. Frequent forms are anxiety, the prevalence of which ranges from 37% [29] to 71.7% [13]. The prevalence of anxiety and apathy is higher in both MSA-P and MSA-C than in healthy controls, whereas that of depression is reduced in MSA-C and relatively consistent in MSA-P [30]. Among frontal lobe behavioral changes in MSA, the most common are apathy and inflexibility, with a frequency between 41% [31] and 57.2% [32]; mild anxiety was reported in 46.8% of MSA patients, and moderate to severe forms were reported in 24.9% [13]. Impaired attention is more frequent in females than in males [20]. A total of 57.2% of Chinese patients with MSA showed moderate frontal behavior changes [32]. Fatigue was prevalent in early-stage MSA, ranging from 28.7% to 64.9%. It increased and remained persistent over time, but was not associated with the severity of motor symptoms [33,34]. Young age and a high anxiety score are associated with fatigue in MSA-P [34]. The prevalence of clinical RBD in MSA patients ranges from 68.8% [27] to 90.2% [14,35,36,37], whereas impulse control disorders and OCD in MSA are less prevalent (13.3%) [16]. Impaired control and disruptive behavioral disorders are exceedingly rare in MSA [38], while attention-deficit/hyperactivity disorder (ADHD), to the best of our knowledge, has never been observed in association with MSA.

## 4. Neuroimaging Findings

### 4.1. Structural Changes

MRI studies in MSA show progressive atrophy of the cerebral cortex, cerebellar white matter (WM), and putamen [39]. Earlier studies reported atrophy of the caudate nucleus and ventral striatum in MSA with affective symptoms [17] (Table 2). Cortical thinning in the bilateral fronto-cingulate cortex, left parietal cortex and left amygdala, and widespread fronto-striatal WM tract fractional anisotropy reduction contribute to executive dysfunction in MSA-P [19], while higher anxiety scores are associated with reduced volume in the bilateral anterior cingulate cortex, precuneus, left amygdala, and bilateral cerebellar tonsils [40].

Apathy and depression in MSA are associated with cortical thinning in the fronto-temporal regions, atrophy of subcortical areas [42], and involvement of structures that links the prefrontal cortex with the limbic system [43]. Changes in functional connectivity (FC) between the nucleus accumbens and dorsal anterior cingulate cortex are associated with apathy [44].

RBD patients show hypointensity of the basal ganglia similar to that in MSA [49] and its specific structural abnormalities (i.e., hot cross-bun sign, putaminal rim, and cerebellar atrophy) [37]. Major neuronal loss in the caudate nucleus, ventral striatum, and parts of the orbitofrontal and limbic circuits may be responsible for anxiety and the blunted affect, which are not responsive to levodopa therapy in MSA [17]. The amygdala is known as a central region in the anxiety and fear circuitry and, therefore, may contribute to the high prevalence of anxiety in synucleinopathies. Studies in animal models showed αSyn pathology in the amygdala associated with alterations of anxiety [50]. It is also related to cortical thinning in the bilateral fronto-cingulate and left parietal cortices [40].

### 4.2. Network Disorders (See Figure 1)

Functional (f) MRI showed that anxiety is associated with increased FC between the amygdala, the orbitofrontal and parietal cortex, the precuneus, and the medial temporal cortex, while increased severity of anxiety is associated with decreased FC between the amygdala and dorsolateral prefrontal cortex, between the striatum and orbitofrontal cortex, and between the orbitofrontal cortex and dorsolateral prefrontal cortex [41]. Moreover, there is stronger FC between the orbitofrontal and temporal cortex and between the striatum, temporal cortex, and cingulate cortex. Increased anxiety is associated with lower FC between the amygdala and dorsolateral prefrontal cortex, and between the orbitofrontal and dorsolateral prefrontal cortex [41]. Anxiety is also associated with changes in the limbic cortico–striato–thalamocortical circuit and with reduced metabolism in the orbitofrontal, dorsolateral and ventrolateral prefrontal and cingulate cortices as well as in striatum [41].

MSA-P patients show decreased connectivity between dorsolateral prefrontal cortex and cerebellar dentate nucleus in addition to disordered striato–thalamocortical networks and enhanced FC between the dentate nucleus and posterior cingulate cortex, which may be significantly associated with executive control and emotional processes [51]. Impaired attention has been related to the dorsolateral prefrontal cortex of the caudate–thalamo–frontal cortex system [23,52].

**Figure 1:**
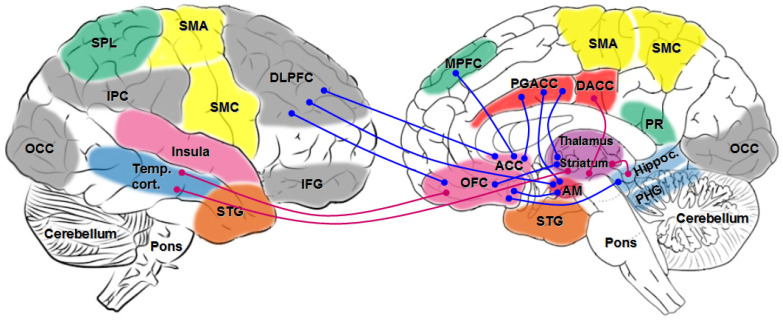
Schematic overview of major network connectivities in behavioral changes in MSA. Functional connectivity: blue lines: decreased, red lines: increased. AM: amygdala; ACC: anterior cingulate gyrus; DACC: dorsal anterior cingulate gyrus; DLPFC: dorsolateral prefrontal cortex; IFG: inferior frontal gyrus; IPC: inferior parietal cortex; MPFC: medial prefrontal cortex; OCC: occipital; OFC: orbital prefrontal cortex; PGACC: perigenual anterior cingulate cortex; PHG: parahippocampal gyrus; PR: precuneus; SMA: supplementary motor cortex; SMC: sensorimotor cortex; SPL: superior parietal lobule; STG: superior temporal gyrus; Temp. cort.: temporal cortex.

### 4.3. Metabolic and Neuromodulatory Changes

[^18^F]-FDG-PET studies showed reduced metabolism in the bilateral dorsolateral prefrontal cortex, together with alterations in corticostriatal WM integrity associated with executive dysfunction that is prominent in MSA-P [19]. Significantly reduced metabolism is found in putamen and caudate nucleus together with increased microglial activation, indicating progression of nigrostriatal dysfunction in RBD patients [46].

Anatomical and functional changes in the amygdala and reduced dopaminergic and noradrenergic activities in the striatum, thalamus, and locus ceruleus, as well as reduced serotonergic activity in the thalamus, are associated with anxiety [41]. On the other hand, lesions of brainstem nuclei, such as the locus ceruleus or raphe nucleus, may promote dysfunction of the cortico–striato–thalamocortical circuit, while other structures, like the subthalamic nucleus, ventral tegmental area, periaqueductal gray matter, and the raphe nuclei, are suggested to be involved in fear and anxiety disorders.

^18^F-fluoroethoxybenzovesamicol (FEOBV) PET studies in RBD from prodromal MSA showed increased FEOBV uptake in specific brainstem areas (bulbar-reticular formation, pontine ceruleus/subceruleus complex, periaqueductal gray, and mesopontine cholinergic nuclei), orbitofrontal, anterior cingulate, and paracentral cortex, indicating increased cholinergic innervation in multiple brain areas. These changes suggest a compensatory cholinergic upregulation in association with the initial phases of neurodegeneration leading to MSA [47,48].

Neuropsychological tests correlated with alterations in corticostriatal WM integrity and reduced metabolism in the bilateral dorsolateral inferior frontal cortex [19]. Compulsive behavior is related to dysfunctions in fronto-striatal systems, including the orbitofrontal, prefrontal, anterior cingulate, and insular cortices and their connections with the basal ganglia [45].

## 5. Pathogenic Mechanisms

In comparison to recent progress in understanding the basic mechanisms of cellular and molecular mechanisms of neurodegeneration in MSA [53], our knowledge about the pathogenesis of behavioral changes in this disorder is limited, depending on recent neuroimaging findings, while specific neuropathological data are not available. Studies in animal models reported αSyn pathology in the amygdala, a central region in the anxiety and fear circuitry, that may contribute to the high prevalence of anxiety in MSA. It is obviously not a bystander symptom in this disorder and other synucleinopathies but reflects early pathogenic mechanisms in the cortico-limbic system which may contribute to disease progression [50]. Neuroinflammatory mechanisms, an essential factor in the pathogenesis of MSA, due to activation of the complement pathway by aggregation of αSyn [54] induce cell toxicity and microglial activation [53,55]. These changes induce neuronal loss in cerebral gray matter (cortex. basal ganglia and brainstem) and microlesions in cerebral WM, which may be responsible for the disruption of essential brain networks. However, due to the lack of relevant clinico-pathological studies, to the best of our knowledge, no definite data about the relevance of neuroinflammatory lesions in MSA for the development of behavioral changes (as well as of cognitive impairment) in MSA are available.

Anxiety and apathy in PD are associated with bilateral fronto-cingulate and parietal cortex atrophy, disordered FC between nucleus accumbens and dorsal anterior cingulate cortex, and higher internetwork resting state FC between the fear and salience network [40,44]. Widespread fronto-striatal WM tract reduction in fractional anisotropy and reduced metabolism in bilateral dorsolateral prefrontal cortex are seen in MSA-P with executive dysfunction [19].

After levodopa therapy, the affective disorders in MSA did not change, which might have been due to neuronal loss in the caudate nucleus and ventral striatum that are part of the lateral orbitofrontal and limbic circuits [17], while unusual compulsive behaviors related to dopamine agonist therapy in MSA suggest that a broad spectrum of psychopathology may occur in this context [56]. The behavior deficit profile of a partial double-lesion rat model mimicked that of early stage in human MSA-P [57], which was also seen in a mouse model of MSA [58].

Isolated RBD is associated with significant cerebral hypometabolism in occipitoparietal and cerebellar regions [59], as well as with a significant reduction in ^18^F-DOPA Ki (^18^F-DOPA-PET scan) in the striatum and widespread microglial activation in substantia nigra indicating profound nigrostriatal dysfunction [46]. Increased cholinergic innervation in multiple brain areas, in particular in the mesopontine area and paracentral cortex, was seen in isolated RBD from prodromal MSA [47].

In conclusion, different combinations of brain circuit disorders that are related to complex pathobiological mechanisms underlie the different behavioral manifestations in MSA.

## 6. Therapeutic Implications

There is no treatment to stop or retard the development of neurodegeneration in MSA and other synucleinopathies and only little chance to influence the development of disease-related behavior disorders. Reversal of behavioral abnormalities by fetal allografts has been reported in a rat model of striatonigral degeneration, suggesting that recovery was due to diffuse dopamine release [60]. Unlike PD, to the best of our knowledge, no such methods have been implemented or tested in human MSA patients.

Depression, anxiety and impulse control disorders may benefit from optimization of dopaminergic therapy or removal of dopamine agonists. Among the currently used symptomatic interventions are antidepressant drugs, including serotonin reuptake inhibitors (SSRIs), serotonin–noradrenaline reuptake inhibitors (SNRIs) and tricyclic antidepressants, but their efficacy is insufficient. In an open-label, non-controlled study, tandospirone, a 5-HT1A agonist that is commonly used in the treatment of anxiety disorders, was more effective in improving depression/anxiety than escitalopram in MSA-C patients [61]. Alternatively, cognitive behavioral therapy (CBT), psychotherapy, physical activity, or exercise may reduce some behavioral symptoms, including anxiety and stress [62]. Treatment options for OCD include pharmacotherapy with SSRIs, requiring higher doses than for other anxiety disorders or major depression. In treatment-resistant OCD, antipsychotic agents like haloperidol, risperdone, olanzapine, or quetiapine may have some efficacy. In conclusion, a person-specific combination of pharmacotherapy and other treatment modalities should be screened and validated in order to obtain the best possible results.

## 7. Conclusions

MSA is a rare oligodendroneuronal α-synucleinopathy characterized by neurodegeneration in striatonigral, olivocerebellar, and multiple other central nervous system regions, which causes complex cumulative motor and non-motor disability, including multiple behavioral disorders as early and pervasive features. Biological hallmarks of the progressing disorder are aggregation and spreading of misfolded αSyn and αSyn strain specificity inducing progressive atrophy of putamen, pons, cerebellar cortex and WM [39], synaptic dysfunction, aberrant proteostasis, iron dyshomeostasis, neuroinflammation, mitochondrial dysfunction, and other cell-specific changes that are relevant to neuronal death and fast progression of MSA [53,63,64].

MSA is frequently associated not only with cognitive impairment and depression, but with a variety of behavioral changes that manifest early and progress with advancing disease, negatively influencing the patients’ quality of life. Besides RBD, a frequent early symptom of MSA, anxiety, impulse control disorders and, less frequently, OCD are the essential behavioral symptoms that are highly variable in prevalence and clinical presentation. Modern neuroimaging data provided some insight into functional mechanisms of behavioral disorders in MSA. Atrophy of fronto-cingulate gray matter and fronto-striatal WM causing disruption of cortico–striato–thalamo–limbic circuits is responsible for anxiety and other affective symptoms. Executive dysfunctions are related to hypometabolism of the prefrontal cortex and corticostriatal dysfunction. RBD, in addition to nigrostriatal dysfunction, is related to increased cholinergic innervation of multiple brain areas, in particular specific brainstem areas, while disorders of the (pre)fronto-striatal system are essential for OCD.

Since behavior abnormalities—like other neuropsychiatric symptoms—show enormous variability among MSA patients, they are still underdiagnosed and undertreated. In the absence of disease-modifying treatment possibilities, symptomatic interventions—such as antidepressants, cognitive behavioral therapy, and antipsychotic agents—are of limited efficacy. Further studies correlating behavioral syndromes in MSA with both functional neuroimaging and postmortem changes are warranted in order to obtain better insight into the underlying pathogenic mechanisms as a basis for early diagnosis and possible future disease-modifying options of this deleterious disorder.

## Figures and Tables

**Table 1:** Behavior disorders *.

Attention-deficit/hyperactivity disorder (ADHD) Oppositional defiant disorder (ODD) Conduct disorder Anxiety disorder including obsessive compulsive disorder (OCD) Disruptive behavior disorders Emotional disorders

* According to the definitions of the Diagnostic and Statistical Manual of Mental Disorders (DSM-5).

**Table 2:** Neuroimaging findings and clinical correlates in MSA.

Modality	Findings	Clinical Correlates	References
Structural MRI	Atrophy of caudate nucleus and ventral striatum	Anxiety	[17]
	Atrophy of bilateral fronto-cingulate and left parietal cortices	Anxiety	[41]
	Atrophy of amygdala and anterior cingulate cortex	Anxiety	[40]
Functional MRI	Disordered orbital and limbic circuits	Anxiety	[17]
	Decreased FC between amygdala, dorsolateral prefrontal cortex, between striatum and orbitofrontal cortex, orbitofrontal and dorsolateral prefrontal cortex. Increased FC between orbitofrontal and temporal cortex, striatum and temporal cortex, striatum, and cingulate cortex.	Anxiety	[41]
	Increased FC between amygdala, anterior cingulate cortex and hippocampus, striatum—medial prefrontal cortex. Reduced FC between lateral orbitofrontal cortex—hippocampus—amygdala. Reduced FC in cortico-striatal thalamocortical circuit. Reduced dopaminergic, noradrenergic and serotonergic activity in striatum, thalamus and locus ceruleus	Anxiety	[40]
	Atrophy of fronto-temporal cortex and subcortical areas	Apathy	[42]
	Reduced fronto-striatal WM tract	Anxiety	[19]
	Disordered link between prefrontal cortex and limbic system	Apathy	[43]
	Disordered FC between accumbens and dorsal anterior cingulate cortex	Apathy	[44]
[^18^F]-FDG-PET	Dysfunction in fronto-striatal system (orbitofrontal, prefrontal, anterior cingulate and insular cortices)	Compulsive behavior	[45]
[^18^F]-DOPA-PET	Reduced metabolism in bilateral dorsolateral prefrontal cortex; alterations in corticostriatal WM integrity	Executive dysfunction	[19]
[^18^F]-FEOBV-PET	Reduced metabolism in putamen and caudate nucleus	RBD	[46]
	Increased cholinergic innervation in multiple brainstem areas/pontine ceruleus/subceruleus complex, bulbar reticular formation, periaqueductal gray matter	RBD	[47,48]

FC: functional connectivity; WM: white matter; RBD: REM sleep behavioral disorders.

## Abbreviations

MSA	Multiple system atrophy.
αSyn	α-Synuclein.
MSA-P	Multiple system atrophy Parkinsonian variant.
MSA-C	Multiple system atrophy cerebellar variant.
PD	Parkinson disease.
RBD	REM sleep behavioral disorders.
OCD	Obsessive compulsive disorders.
FC	Functional connectivity.
WM	White matter.
